# Digitally Enhanced Routine Outcome Monitoring in Italian Psychotherapy: Protocol for a Longitudinal Implementation Study

**DOI:** 10.2196/82837

**Published:** 2026-03-02

**Authors:** Mattia Vincenzo Olive, Antonino La Tona, Vivian Ostwald, Agostino Brugnera, Gianluca Lo Coco, Angelo Compare, Dana Tzur Bitan, Cristina Masella

**Affiliations:** 1 Department of Management, Economics and Industrial Engineering, Politecnico di Milano, Via Raffaele Lambruschini 4b, Milan, 20156, Italy, 39 3488676027; 2 Department of Human and Social Sciences, Università degli Studi di Bergamo, Bergamo, Italy; 3 Department of Psychology and Educational Sciences, Università di Palermo, Palermo, Italy; 4 Department of Community Mental Health, University of Haifa, Haifa, Israel

**Keywords:** implementation, psychotherapy, routine outcome monitoring, ROM, Consolidated Framework for Implementation Research, CFIR, protocol, Italy

## Abstract

**Background:**

Routine outcome monitoring (ROM) is an evidence-based methodology in psychotherapy that supports clinical decision-making through the standardized and systematic collection of patient-reported outcome data. Although its benefits are well established, ROM remains poorly integrated into routine psychotherapeutic practice in many countries, including Italy. Structural fragmentation of services, limited digital infrastructure, and cultural resistance within the profession hinder the adoption of data-informed clinical practices.

**Objective:**

This study aims to investigate the implementation of a digital platform designed to support ROM in Italian psychotherapy, with a focus on individual, organizational, and contextual factors influencing adoption and integration into everyday clinical practice.

**Methods:**

This is a longitudinal, mixed methods implementation study embedded within the OutProFeed project, the first randomized controlled trial in Italy focused on ROM in psychotherapy. The study is guided by the Consolidated Framework for Implementation Research, which informed the selection of constructs through a structured expert consensus process. Approximately 30 psychotherapists are included across diverse practice settings, therapeutic orientations, and geographic areas. Data will be collected at three time points (T0 baseline, T1 midimplementation at ~6 mo, and T2 final follow-up at ~12 mo). Qualitative data will be generated through semistructured interviews, while quantitative data will be collected using the Evidence-Based Practice Attitude Scale–36 (EBPAS-36). Qualitative and quantitative data will be linked at the individual level to support integrated interpretation of implementation trajectories.

**Results:**

The study received ethical approval from the Ethics Committee of the University of Bergamo in April 2024. Technical setup of the digital platform and preimplementation engagement with participating psychotherapists were conducted between May and November 2025. Baseline (T0) implementation data collection started on a rolling basis in December 2025 and is planned to continue through March 2026. The midimplementation follow-up (T1; approximately 6 mo after individual baseline) is expected to occur between June and October 2026, with the final follow-up (T2; approximately 12 mo after baseline) planned between December 2026 and April 2027. Data analysis will begin after completion of follow-up assessments, with initial findings expected to be submitted for publication in 2027.

**Conclusions:**

This protocol outlines a theory-informed and context-sensitive approach to studying the implementation of digitally supported ROM in psychotherapy. By combining qualitative and quantitative data within a longitudinal design, the study aims to generate insights into the conditions under which digital outcome monitoring may be integrated into routine psychotherapeutic work in fragmented and professionally autonomous health care systems.

## Introduction

Routine outcome monitoring (ROM) is an evidence-based methodology used in psychotherapy to support the continuous assessment and improvement of therapeutic processes through the standardized and systematic collection of clinical data [1-3]. ROM involves the regular administration of validated self-report questionnaires to monitor patient well-being, treatment progress, and the quality of the therapeutic alliance [1-4]. The data collected are typically visualized in graphical form and shared with the psychotherapist and, when appropriate, with the patient, enabling collaborative reflection and timely, data-informed adjustments to the therapeutic plan [3,5].

By integrating a structured feedback loop into clinical practice, ROM fosters greater transparency in the therapeutic relationship and promotes a more individualized and adaptive approach to care [6]. Empirical studies have demonstrated its potential to improve clinical outcomes, increase patient engagement, and detect early signs of stagnation or deterioration in treatment [2,6-8].

Historically, several barriers have limited the adoption of ROM in clinical practice [9]. For instance, paper-based methods often resulted in fragmented and delayed data collection, undermining the timeliness and utility of feedback. The growing availability of digital tools has addressed many of these limitations by enabling real-time data entry, automated analysis, and immediate visualization of results. Digital platforms enhance psychotherapists’ ability to track progress over time, prepare for sessions more effectively, and ensure better continuity of care across encounters [5,10-16].

The American Psychological Association has long advocated the routine use of outcome and process monitoring in psychological care [17]. However, ROM implementation remains limited in many contexts, including Italy [18]. In Italy, this challenge is intensified by the organization of the mental health system: psychotherapy is not included among the services guaranteed by the national health system, resulting in a fragmented landscape dominated by private practice and high variability in theoretical orientations and documentation practices [19-21]. This context poses specific challenges for implementing structured practices such as ROM.

From an international perspective, the Italian case is analytically relevant because it makes visible implementation dynamics that are likely to emerge in many psychotherapy and mental health systems characterized by high professional autonomy, limited institutional coordination, and pluralistic practice arrangements, rather than representing an exceptional context.

This study contributes to bridging the gap between the recognized value of ROM and its limited implementation in real-world psychotherapeutic settings. The OutProFeed project, the first randomized controlled trial in Italy focused on ROM in psychotherapy, evaluates the implementation of digitally supported ROM, enabled by a digital platform (Mindy), designed to support psychotherapists in collecting and using clinical data in compliance with privacy regulations. Mindy is a digital platform that supports the administration of routine outcome measures and provides structured feedback through visual dashboards. Its main features, including the data collection interface, feedback visualization, and security-related design choices, are described in detail in a prior usability study [22].

While grounded in the Italian context, the study also offers insights that may inform implementation efforts in other health systems facing similar structural, technological, or cultural barriers [23-25]. By documenting the implementation process, this research provides a practical foundation for the broader adoption of data-informed practices in psychotherapy [26].

## Methods

### Overview

This study investigates the implementation of digitally supported ROM in Italian psychotherapeutic practice using the Mindy platform. Building on existing international evidence regarding the benefits of ROM in enhancing therapeutic effectiveness, this research focuses on understanding how such a practice can be introduced, adopted, and sustained in a context characterized by a high degree of professional autonomy, heterogeneous clinical approaches, and limited institutional support. The study was designed as a mixed methods implementation study to capture the complexity of this process, addressing not only whether ROM can be used but also how it is actually integrated into psychotherapists’ clinical routines and what contextual factors facilitate or hinder this integration. A combined qualitative and quantitative approach was deemed necessary to generate both explanatory insight and empirical grounding for a robust analysis of implementation dynamics.

### The Consolidated Framework for Implementation Research and the Rationale Behind Its Employment

To guide the design and evaluation of this mixed methods implementation study, we adopted the Consolidated Framework for Implementation Research (CFIR) [27,28], a widely recognized framework to support the systematic analysis of complex implementation processes. CFIR brings together constructs from multiple theoretical models and organizes them into 5 domains: the characteristics of the innovation, the outer setting, the inner setting, the individuals involved, and the implementation process. CFIR was selected not only for its comprehensiveness but because it is theoretically suited to examine implementation in professional domains characterized by high autonomy, weak formal hierarchy, and discretionary practice, such as psychotherapy. Unlike frameworks that focus primarily on organizational readiness or intervention fidelity, CFIR explicitly accommodates the interaction between individual professionals, loosely structured work settings, and evolving implementation processes. This is particularly relevant for psychotherapy, where adoption of structured practices such as ROM does not occur through formal mandates but through negotiated alignment with professional judgment, therapeutic orientations, and everyday clinical routines. CFIR therefore provides a theoretically appropriate lens to analyze implementation as a multilevel and processual phenomenon, in which professional agency, contextual constraints, and characteristics of the innovation jointly shape how digitally supported ROM is interpreted and integrated into practice.

In this study, CFIR was used from the outset to inform the development of the research design, the construction of qualitative and quantitative data collection instruments, and the approach for coding and interpretation of findings. The framework’s adaptability proved particularly valuable in structuring the analysis of an intervention (ROM) via a digital platform, introduced into a highly fragmented and heterogeneous psychotherapeutic system, such as the one operating in Italy. CFIR’s emphasis on capturing the perspectives of those directly involved in the implementation aligns with the study’s focus on psychotherapists as both subjects and agents of change.

The framework is not applied at a single point in time but is used to guide data collection and analysis throughout the full implementation trajectory [29]. This continuous engagement with CFIR supports the identification of context-specific dynamics as they evolve, allowing the study to capture temporal variation and the interaction between individual, organizational, and systemic factors. The structured yet flexible nature of CFIR enhances the study’s explanatory power and supports the development of insights that are both context-sensitive and transferable beyond the Italian setting.

### Implementation Setting

The implementation study is part of the broader *OutProFeed* project, which aims primarily to assess the clinical effectiveness of digitally supported ROM in improving mental health outcomes. Alongside this central objective, the project also includes 2 complementary strands of investigation: one focusing on the usability of the digital platform and the other, addressed in this study, exploring the conditions under which ROM can be successfully adopted and sustained in everyday psychotherapeutic practice. The present work specifically examines the implementation process, taking into account contextual, organizational, and individual-level factors.

The unit of analysis is the individual psychotherapist. This choice reflects the structure of the Italian psychotherapeutic landscape, in which professionals often work autonomously and exercise substantial discretion in adopting new tools and practices [30,31]. By focusing on the psychotherapist as the key actor in the implementation process, the study aims to capture the variability in professional routines, digital readiness, and perceived value of ROM, all of which are expected to influence the integration of digitally supported ROM into routine psychotherapeutic practice.

The study is conducted within the Italian mental health system, where psychotherapy provision is largely fragmented across private practice and under-resourced public services [21,32]. As a result, service provision is predominantly private, with many psychotherapists operating independently and outside institutional structures. Even within the public system, psychotherapy is available only in a limited number of settings, such as family counseling centers and specialized mental health services, which often suffer from limited resources and organizational constraints. This context creates significant variation in how psychotherapy is delivered, regulated, and documented across the country [32,33].

Such heterogeneity offers a unique opportunity to examine implementation in a real-world setting characterized by institutional gaps, inconsistent digital infrastructure, and diverse professional orientations. The insights generated by this study are intended to inform not only national efforts to scale up ROM but also broader international discussions on the conditions needed to support the effective integration of digital tools in mental health care.

### Implementation Outcome

The study targets early-stage implementation at the level of professional practice. The primary implementation outcome is the integration of digitally supported ROM into psychotherapists’ everyday clinical work. Integration is understood as the extent to which ROM is practically taken up, interpreted, and incorporated into routine therapeutic activities over time, rather than as an indicator of clinical effectiveness or system-level diffusion. The study examines this outcome by exploring how psychotherapists engage with the platform, how ROM aligns with professional routines and norms, and how ROM-related activities are incorporated into ongoing clinical practice. This corresponds to early-stage outcomes such as acceptability, appropriateness, and perceived feasibility of digitally supported ROM at the level of professional practice, rather than to clinical effectiveness or system-level adoption. The CFIR framework is used to identify and interpret the individual, organizational, and contextual conditions that shape this implementation process.

In this study, successful implementation is operationalized in processual terms, rather than through predefined performance thresholds. Meaningful implementation is indicated by psychotherapists’ reported incorporation of ROM-related activities into routine clinical work, perceived alignment between ROM and therapeutic practice, and sustained engagement with the digital platform over time. Changes in Evidence-Based Practice Attitude Scale–36 (EBPAS-36) scores are interpreted as complementary indicators of evolving openness toward evidence-based and structured practices, while qualitative data provide the primary basis for assessing how such changes translate into everyday clinical routines. This approach is consistent with early-stage implementation research, where the aim is to understand how practices are integrated and adapted, rather than to quantify adoption or effectiveness.

### CFIR Construct Selection and Consensus Process

Given the extensive range of constructs within the CFIR, a structured and transparent process was developed to identify the constructs most relevant to the implementation of digitally supported ROM in psychotherapy. The objective was to ensure a balance between theoretical completeness and practical applicability, adapting the framework to the specific characteristics of the intervention and its context.

The final set of selected constructs is reported in Table 1. The selection was guided by four exclusion criteria, applied in the following order:

- *Relevance to the Italian context:* Constructs whose variation is primarily driven by national-level or cross-country factors were excluded, given the single-country scope of the study.
- *Accessibility of information to participants:* Constructs requiring information not reasonably accessible to practicing psychotherapists were removed in order to ensure the reliability of the data collected.
- *Empirical support from existing literature:* Constructs that have shown limited relevance in comparable implementation studies were deprioritized, while those consistently associated with implementation outcomes were prioritized.
- *Specificity to the case under study:* Residual elements not covered by the above criteria were considered in light of the characteristics of the platform, the type of clinical practice, and the level of professional autonomy.

**Table 1.** Selected constructs.

Domain and construct	Average rating	Selected in phase 1	Selected in phase 2
Innovation			
Source	1.67		
Evidence base	2		
Relative advantage	6.33	✓	
Adaptability	6.33	✓	
Trialability	1		
Complexity	5.5	✓	
Design	5.5	✓	
Cost	1.67		
Outer setting			
Critical incidents	1		
Local attitudes	1		
Local conditions	1.67		
Partnerships and connections	1.67		
Policies and laws	5		
Financing	1		
External pressure	1		
Societal pressure	1		
Market pressure	1		
Performance measurement pressure	1		
Inner setting			
Structural characteristics	1		
Physical infrastructure	1		
IT infrastructure	1.83		✓
Work infrastructure	1.5		✓
Relational connections	1.5		
Communications	1.5		✓
Culture	2.5		
Human equality-centeredness	1		
Recipient-centeredness	4.17	✓	
Deliverer-centeredness	1.83		✓
Learning-centeredness	4.33	✓	
Tension for change	6		
Compatibility	6.67	✓	
Relative priority	3.83		✓
Incentive system	5		
Mission alignment	6.67	✓	
Available resources	1		
Funding	1.67		
Space	1		
Individuals			
High-level leaders	1		
Mid-level leaders	1		
Opinion leaders	1		
Implementation facilitators	2		✓
Implementation leads	1		
Implementation team members	1		
Other implementation support	1		
Innovation deliverers	1		
Innovation recipient	1		
Need	1.5		
Capability	6.17	✓	
Opportunity	6.33	✓	
Motivation	3.5		✓
Implementation process			
Teaming	1		
Assessing needs	4.83		
Innovation deliverers	1		
Innovation recipient	4.5	✓	
Assessing context	4.83	✓	
Planning	1		
Tailoring strategies	1		
Engaging	2		
Innovation deliverers (F1)	1.67		
Innovation recipient (F2)	4.33		
Doing	2.17		
Reflecting and evaluating	2		
Implementation (H1)	1		
Innovation (H2)	5.67	✓	
Adapting	3.5		✓

The evaluation process involved 9 contributors, selected for their complementary expertise:

- Three experts in digital health and management of digital transformation, affiliated with academic institutions and research centers
- Six professionals with backgrounds in clinical psychology and psychotherapy, with practical experience in the field and familiarity with the intervention

A two-step consensus process was then conducted:

1. Phase 1—Individual rating: each contributor independently assessed the relevance of each CFIR construct using a 7-point Likert scale (1=low relevance; 7=high relevance) and provided optional qualitative comments to support their evaluations.
2. Phase 2—Plenary discussion: two consensus meetings were held to discuss constructs with intermediate or divergent ratings. This collaborative phase allowed for the reconsideration of constructs based on disciplinary perspectives and case-specific insights.

Following consensus, each selected construct was associated with a specific interview item. These items were iteratively revised to ensure conceptual consistency with the framework, clarity of language, and clinical appropriateness. The resulting model supports a focused and theoretically grounded analysis of the implementation process, adapted to the structure of psychotherapy practice in Italy.

### Data Collection and Analysis

#### Overview

Data will be collected over the study period through a mixed methods design combining semistructured interviews and a validated questionnaire (EBPAS-36). The objective is to investigate how psychotherapists engage with the digital platform *Mindy* and to identify the factors that facilitate or hinder the implementation of digitally supported ROM across different phases of adoption.

**Figure 1.**
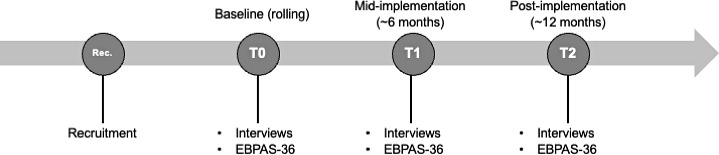
Study design and data collection timeline (rolling baseline with longitudinal follow-up). EBPAS-36: Evidence-Based Practice Attitude Scale–36.

Data collection follows a rolling baseline design (Figure 1). After enrollment, each participant completes a baseline assessment (T0) on a rolling basis between December 2025 and March 2026, with follow-up assessments scheduled approximately 6 months (T1) and 12 months (T2) after their individual baseline. The midimplementation follow-up (T1) is expected to occur between June and October 2026 and the final follow-up (T2) between December 2026 and April 2027. Because enrollment occurs on a rolling basis, calendar dates for follow-ups differ across participants and partially overlap.

#### Qualitative Data Collection

Semistructured interviews will be conducted with a sample of around 30 psychotherapists, selected to reflect the diversity of the Italian context in terms of employment setting (private, public, freelance), therapeutic orientation, and geographical location. Recruitment is carried out within the broader OutProFeed project through established collaborations with psychotherapy training schools and their professional networks, complemented by outreach to practitioners in both private practice and organizational settings. The research team monitors the evolving sample composition during enrollment to avoid overrepresentation of specific profiles (eg, a single orientation or geographic area) and to ensure coverage across the predefined variation dimensions. This recruitment approach is intended to reduce selection bias while remaining consistent with the study’s purposive, maximum-variation design. While an initial target of approximately 30 participants is set, the final sample size will be determined during the research process. Data collection will continue until all these key dimensions are represented and data saturation is reached.

The target sample of approximately 30 psychotherapists is grounded in the structural and professional heterogeneity of psychotherapy practice in Italy. In particular, the sampling strategy is designed to ensure coverage of the main psychotherapeutic orientations currently practiced in the Italian context, which represent distinct clinical traditions and professional cultures that may shape how ROM is interpreted and integrated into everyday practice. The study aims to include psychotherapists from at least five widely adopted approaches (eg, cognitive-behavioral, psychodynamic, systemic-relational, humanistic, and integrative orientations).

Within this framework, participants are purposively recruited to also reflect variation in employment arrangements (private practice and organizational settings) and geographic macro-areas (North, Center, South/Islands). With a target of approximately 30 participants, the study can include multiple psychotherapists within each major orientation while preserving sufficient diversity across organizational and regional contexts. This sample size supports comparative, process-oriented analysis of implementation trajectories across different professional logics, without aiming for statistical representativeness.

A purposive subsample will be followed longitudinally across T0, T1 (~6 mo), and T2 (~12 mo) to enable within-case process tracing; all participants will be invited at each wave. Meaningful longitudinal analyses will be conducted for participants with repeated observations across waves, with attrition explicitly documented and reported.

The interview guide, based on the CFIR constructs selected during the consensus process, is included in Multimedia Appendix 1. It includes approximately 16 questions for psychotherapists in private practice and 20 questions for those employed in organizations. Interviews will be conducted individually in Italian via Microsoft Teams, in quiet and distraction-free settings. Each session will be led by a pair of researchers, one with clinical expertise in psychotherapy and one with a background in digital transformation, to ensure both contextual sensitivity and methodological coherence.

With informed consent, all interviews will be audio-recorded and transcribed verbatim. Interviewers will adopt an open, nondirective style to encourage participants to reflect freely on their experience. The average duration of interviews is expected to be between 25 and 30 minutes.

#### Qualitative Data Analysis

Thematic analysis will be applied to the interview transcripts, following the methodology proposed by Braun and Clarke [34]. The CFIR framework will guide the coding process and the organization of emerging themes [35]. Analysis will be conducted using Excel as an analytic workspace. The process will begin with close reading and familiarization, followed by a deductive coding phase that maps participants’ responses onto the relevant CFIR domains and constructs. This approach supports a structured interpretation of barriers and facilitators, while also allowing room for emerging contextual elements.

#### Quantitative Data Collection and Analysis

In parallel with each interview, participants will complete the EBPAS-36, a validated instrument that assesses attitudes toward adopting evidence-based practices. In line with the rolling baseline design, T1 and T2 are scheduled approximately 6 and 12 months after each participant’s baseline assessment.

The EBPAS-36 consists of 36 items rated on a 5-point Likert scale and includes multiple subscales. Descriptive statistics will be calculated for each wave of data collection, including mean scores and SDs. Negative subscale items will be reverse-coded so that higher scores consistently indicate more positive attitudes toward innovation and structured clinical practices.

Repeated measures will be analyzed using a descriptive longitudinal approach. For participants providing data at multiple time points, changes in EBPAS-36 total and subscale scores will be explored within individuals, focusing on the direction and magnitude of change across waves. Where appropriate and depending on data completeness and distributional assumptions, nonparametric paired comparisons (eg, Wilcoxon signed rank tests) may be used to explore differences between time points. These analyses are intended to be exploratory and supportive of the qualitative findings, rather than hypothesis testing.

Quantitative data will be linked to the corresponding qualitative data at the level of individual participants. This integration will enable a dual-layered analysis of each psychotherapist’s experience, combining narrative accounts with attitudinal profiles. Although not intended as a formal triangulation, this approach will enhance the interpretive depth of the study and contribute to a deeper understanding of implementation trajectories over time.

#### Data Integration

Qualitative and quantitative data are integrated at the level of individual participants using a convergent, case-oriented mixed methods approach. For each psychotherapist, interview data constitute the primary source for reconstructing the implementation process and its evolution over time. EBPAS-36 scores are used as complementary information to characterize psychotherapists’ attitudinal positioning toward evidence-based practices at each time point and to support the interpretation of qualitative changes or continuities. Integration, therefore, occurs through iterative comparison within cases, examining how shifts in narratives of ROM integration relate to stable or changing attitudinal profiles. This strategy prioritizes explanatory coherence over formal triangulation and is aligned with the study’s focus on understanding implementation as a situated professional process.

### Pilot Test

A pilot phase was conducted with 3 psychotherapists to assess the adequacy and feasibility of the data collection protocol and to support its refinement prior to the main study. This pilot phase was explicitly conceived as a feasibility and protocol-refinement step and not as a source of empirical findings or preliminary implementation results. It provided a pragmatic opportunity to test the interview guide developed around the selected CFIR constructs (Table 1) and to assess the clarity, flow, and acceptability of the interview structure and language.

The interview guide used during the pilot is available in Multimedia Appendix 2. An initial version had been prepared based on the selected constructs. Insights from the pilot led to minor procedural refinements, primarily concerning the sequencing and phrasing of questions to facilitate a more natural conversational flow consistent with clinical interaction. The substantive content of the questions was not altered, and all targeted constructs were retained.

Quantitative data were not collected at this stage. The EBPAS-36 questionnaire, which will be administered in the main phase of the study, is a validated instrument that requires a sufficiently large and diverse sample to yield meaningful results. Given the feasibility-oriented purpose of the pilot and the small number of participants involved, its administration at this stage was not considered appropriate.

### Ethical Considerations

The study has been reviewed and approved by the Committee for Integrity and Research Ethics of the University of Bergamo (2024_04_08) in compliance with national regulations and the Declaration of Helsinki. All participants are informed about the aims of the research, the voluntary nature of their participation, and the possibility to withdraw at any time without consequences.

Before each interview, participants receive an information sheet and are asked to provide written informed consent, including permission to record the session. Consent forms are stored securely and separately from the collected data. Interviews are conducted in a confidential setting using secure online platforms. Audio recordings are transcribed verbatim and anonymized to ensure participant privacy.

All personal identifiers are removed or replaced with pseudonyms during transcription. Data are stored in encrypted, password-protected institutional folders accessible only to the research team. Files are regularly backed up and managed in accordance with institutional data protection policies and the European General Data Protection Regulation.

For psychotherapists operating in private practice and using the digital platform, the implementation study does not involve access to identifiable patient-level clinical data. The research team does not access or extract raw clinical content entered into the platform. Data collected for research purposes focus exclusively on psychotherapists’ experiences, attitudes, and implementation processes and are analytically and technically separated from any clinical documentation generated within routine practice.

Quantitative data collected through the EBPAS-36 questionnaire are similarly anonymized and securely stored. The integration of qualitative and quantitative data at the individual level is performed using anonymized study codes, ensuring that no identifying information is used in the analysis or dissemination of findings.

Dissemination of results will take place in aggregated form only, and no individual participant will be identifiable in publications or presentations. Participants are also informed that the results may contribute to academic and policy-oriented outputs aimed at improving clinical practice in the field of psychotherapy. No participant compensation is provided.

## Results

This implementation study is conducted within the broader OutProFeed project, which received ethical approval from the Ethics Committee of the University of Bergamo in April 2024 (protocol number 2024_04_08); the present work is a predefined substudy covered by this approval. Technical setup of the digital platform and engagement with participating psychotherapists were conducted between May and November 2025. Baseline (T0) implementation data collection started on a rolling basis in December 2025 and is planned to continue through March 2026. The midimplementation follow-up (T1; approximately 6 mo after individual baseline) is expected to take place between June and October 2026, with the final follow-up (T2; approximately 12 mo after baseline) planned between December 2026 and April 2027. As of the time of manuscript submission, no implementation outcome analyses have been completed.

## Discussion

### Main Contributions and Anticipated Insights

This implementation study is designed to generate practice-relevant insights into how digitally supported ROM is incorporated into routine psychotherapy work in a fragmented and professionally autonomous care system. Specifically, we expect to identify (1) the implementation mechanisms through which psychotherapists interpret, negotiate, and integrate ROM into existing clinical routines, (2) the contextual and organizational conditions that enable or constrain this integration, and (3) how these dynamics vary across therapeutic orientations and practice arrangements. These anticipated contributions are implementation-focused and do not concern clinical effectiveness, which is addressed within the broader OutProFeed trial.

ROM and feedback-informed approaches are supported by a robust body of evidence demonstrating their potential to improve clinical outcomes, patient engagement, and early detection of treatment deterioration [2,6,7]. Despite this evidence, the integration of ROM into routine psychotherapeutic practice remains uneven, particularly in settings where its use requires sustained changes to psychotherapists’ workflow, documentation practices, and interaction with patients [3,5,9]. Research on the digital transformation of mental health services further suggests that digital platforms can lower some practical barriers to ROM adoption but do not in themselves resolve deeper professional and organizational challenges related to implementation [10]. In this context, the present protocol explicitly focuses on implementation as a professional and organizational process, rather than on clinical effectiveness, which is addressed within the broader OutProFeed trial.

Despite the evidence base, ROM adoption in psychotherapy is often hindered by barriers that are well documented in implementation and measurement-based care research. These include psychotherapists’ concerns that structured monitoring may constrain clinical discretion or undermine therapeutic work, uncertainty about data ownership and potential “surveillance” uses of outcome data, and perceived workload associated with repeated administration and review of measures [3,5,6,9]. In digitally mediated models of care, technology can reduce friction in data capture and feedback, but it can also amplify sensitivities related to confidentiality, governance, and professional autonomy if the tool is perceived as externally imposed or poorly aligned with clinical routines [10]. These barriers are expected to be particularly salient in fragmented systems dominated by independent private practice, such as Italy, where formal organizational mandates and shared infrastructures are limited [18,21].

### Relation to Prior Implementation Research

From an implementation science perspective, this study responds to recent calls to strengthen mental health implementation research by attending more closely to contextual variation, professional practices, and the dynamics of early-stage implementation [26]. The Italian psychotherapy ecosystem offers a particularly salient empirical setting for this purpose. Psychotherapy in Italy is characterized by high professional autonomy, strong theoretical pluralism, and limited institutional coordination, with service provision largely fragmented across private practice and under-resourced public services [18,21]. In such a context, the uptake of structured monitoring practices such as ROM is likely to depend less on formal mandates or technical availability and more on how these practices are perceived to fit with psychotherapists’ professional identities, therapeutic orientations, and everyday clinical routines.

Consistent with this view, the study conceptualizes implementation as an evolving process of professional integration rather than as a binary outcome of adoption versus nonadoption. Preliminary insights from the pilot phase suggest that variability across therapeutic orientations and practice arrangements is not incidental but central to understanding how ROM is interpreted and enacted in practice. This aligns with implementation research emphasizing nonlinear and processual models of change, in which innovations are adapted, negotiated, and selectively incorporated into routine work over time. The use of the CFIR provides a structured analytical lens to identify multilevel determinants of these processes while enabling comparison with prior implementation studies in digital mental health and psychotherapy [27,28].

### Methodological Strengths and Limitations

Methodologically, the longitudinal mixed methods design is intended to capture temporal dynamics in psychotherapists’ engagement with ROM, addressing limitations of cross-sectional approaches that treat attitudes and practices as static predictors. Qualitative interviews serve as the primary source for reconstructing implementation trajectories, while repeated measurement of attitudes toward evidence-based practices using the EBPAS-36 offers complementary insight into how openness to evidence-based approaches may relate to evolving experiences of ROM integration. In line with the study’s implementation focus, these data are used for explanatory interpretation rather than for testing causal models or estimating effectiveness. Two limitations should be noted. First, the purposive sample and the focus on one national context support analytic transferability rather than statistical generalizability. Second, the reliance on self-reported accounts and repeated interviews may be subject to social desirability and attrition over time, which we will document and consider in interpretation.

### Dissemination

Following completion of data collection, findings will be disseminated through peer-reviewed publications, academic conferences, and stakeholder-facing dissemination activities, contributing to ongoing debates on how data-informed practices can be embedded into routine psychotherapeutic care in contexts characterized by professional autonomy and organizational fragmentation [26].

## Supplementary material

10.2196/82837 Multimedia Appendix 1 Interview protocol.

10.2196/82837 Multimedia Appendix 2 Summary of insights from the pilot interviews by Consolidated Framework for Implementation Research domain.

## Abbreviations

CFIR: Consolidated Framework for Implementation Research
EBPAS-36: Evidence-Based Practice Attitude Scale–36
ROM: routine outcome monitoring
