# Supplementary material for: Digitally Enhanced Routine Outcome Monitoring in Italian Psychotherapy: Protocol for a Longitudinal Implementation Study
Source: JMIR Res Protoc. 2026 Mar 2;15:e82837. doi: 10.2196/82837 (PMC12954688; doi:10.2196/82837)
Supplement: Multimedia Appendix 1 [file resprot-v15-e82837-s001.docx]

**Supplementary File 1 – Interview protocol**

The interview begins with a brief explanation of the study objectives, including the use of Routine Outcome Monitoring (ROM) and the role of the digital platform *Mindy*. All participants are required to have used the platform recently.

The following questions are asked during each interview, unless otherwise indicated.

1. What is your psychological orientation or school of psychotherapy, and which therapeutic approach do you most frequently apply in your clinical work? (behavioral, cognitive, dialectical, interpersonal, person-centered, psychodynamic, experiential, etc.)

*Only asked at the first interview.*

1. How much does the opinion of your colleagues or your professional association/school influence your view on this topic? Do you feel your opinion is influenced by theirs?
   *Only asked at the first interview.*
2. Have you ever felt the need to reduce the burden of administrative tasks or the overwhelming number of messages, such as those received via WhatsApp?
   *Only asked at the first interview.*
3. How often do you use digital tools in your daily life and in your professional activities? Do you find them easy to use?

*Only asked at the first interview.*

1. Do you use digital tools to manage your work schedule in daily practice?
   *Only asked at the first interview.*
2. Do you think that using Mindy for ROM fits into your typical daily workflow?
3. Do you think that using ROM through Mindy offers you any advantages compared to not using it?
4. What do you see as potential disadvantages of using Mindy, or what might make you stop using the platform?
5. How important is it for a platform to be customizable? To what extent do you think Mindy meets this criterion?
6. Do you think that ROM through Mindy is customizable enough to meet your needs?
7. Do you think using ROM through Mindy is complicated in your day-to-day clinical practice?
8. Are you able to navigate Mindy without difficulty when using it for ROM?
9. Do you have access to a computer or tablet suitable for using Mindy?
10. *Only for participants working within an organization (not for freelancers)*

- Would your manager approve of you using ROM through Mindy in your clinical work?
- Does using ROM through Mindy conflict with the shared values or policies of your organization, for example regarding patient care, therapist support, safety, or quality improvement?
- Is the use of ROM through Mindy aligned with your organization’s goals? Would there be any incentives, formal or informal, for adopting such a system?
- Does using ROM through Mindy affect interactions among colleagues, for instance in terms of information exchange or collaboration?

1. Do you feel that you have the time and autonomy needed to effectively integrate a digital ROM system into your therapeutic practice?
2. Does using ROM through Mindy have any impact on your personal or professional goals?
3. Do you foresee any barriers or facilitators to the continued implementation of ROM through Mindy in your clinical practice?
4. What do you consider to be the key success factors of ROM through Mindy, and what aspects do you think could be improved?
