# Supplementary material for: Digitally Enhanced Routine Outcome Monitoring in Italian Psychotherapy: Protocol for a Longitudinal Implementation Study
Source: JMIR Res Protoc. 2026 Mar 2;15:e82837. doi: 10.2196/82837 (PMC12954688; doi:10.2196/82837)
Supplement: Multimedia Appendix 2 [file resprot-v15-e82837-s002.docx]

**Supplementary File 2 – Summary of insights from the pilot interviews by CFIR domain**

| **CFIR Domain** | **Preliminary Observations** |
| --- | --- |
| **Innovation** | Therapists appreciated the structured nature of ROM and the clarity of the platform’s graphical interface. However, the lack of flexibility in workflow and limited customization options may reduce its suitability for different therapeutic styles. |
| **Inner Setting** | Digital infrastructure and a positive orientation toward innovation supported early engagement. Public sector environments appeared more constrained due to bureaucratic processes, absence of legal guidance, and cultural resistance to change. |
| **Individuals** | Therapists' motivation was closely tied to their perception of the platform’s usability and clinical utility. Familiarity with digital tools was an enabling factor, whereas limited autonomy and digital competence emerged as potential barriers. |
| **Implementation Process** | Priorities included technical reliability, legal clarity, and alignment with existing workflows. psychotherapists emphasized the importance of an iterative approach that incorporates user feedback throughout the implementation process. |
